# Multiwalled Carbon Nanotube Reinforced Electrospun Biodegradable Polybutylene Succinate: Electromagnetic Shielding, Thermal and Mechanical Properties

**DOI:** 10.3390/polym17172381

**Published:** 2025-08-31

**Authors:** Usman Saeed, Hisham Bamufleh, Abdulrahim Alzahrani, Aqeel Ahmad Taimoor, Sami Ullah Rather, Hesham Alhumade, Walid M. Alalayah, Hamad AlTuraif

**Affiliations:** Chemical & Materials Engineering Department, Faculty of Engineering, King Abdulaziz University, Jeddah 21589, Saudi Arabia; hbamufleh@kau.edu.sa (H.B.); azahrani@kau.edu.sa (A.A.); aataimoor@kau.edu.sa (A.A.T.); halhumade@kau.edu.sa (H.A.); walalayah@kau.edu.sa (W.M.A.); hturaif@kau.edu.sa (H.A.)

**Keywords:** biodegradable, polybutylene succinate (PBS), multiwalled carbon nanotubes (MWCNT), electrospinning

## Abstract

An environmentally friendly biodegradable and flexible polymer with exceptional mechanical, thermal and electromagnetic interference shielding is urgently needed to reduce environmental pollutants and electromagnetic waves to preserve human health. The paper presents our study where we developed biodegradable electrospun nanocomposite by employing polybutylene succinate (PBS) with multiwalled carbon nanotubes (MWCNTs). The crystallization temperature Tc and melting temperature Tm of electrospun PBS/MWCNT composites with 3 wt% of MWCNTs was increased noticeably by 4 °C and 5 °C. The tensile strength increased by about 2.61 ± 0.15 MPA and the elastic modulus increased by about 0.72 ± 0.02 GPa with the addition of 3% MWCNT in polybutylene succinate. The increase in MWCNT content from 0.5 to 3 wt% led to an enhanced storage modulus and electrical properties 5 to 8 times higher in comparison to PBS. Moreover, the MWCNT was tested in different concentrations in PBS for electromagnetic interference shielding (EMI) and the most applicable results were obtained when the MWCNT was 3% which is capable of providing 25.5 db EMI shielding efficiency. The percolation threshold capability of PBS/MWCNT electrospun nanocomposites was 0.94 wt% and has significant entanglement of the MWCNTs and MWCNT network in the PBS matrix for conductive pathways. The study offers a viable process for creating an electrospun PBS/MWCNT composite that is lightweight, biodegradable and has exceptional electromagnetic shielding capabilities.

## 1. Introduction

The use of biodegradable electrospun polymers has grown in popularity in recent decades due to the environmental pollution issues brought on by excessive fossil fuel consumption and the biological health issues brought on by microplastics. Polymers like polybutylene succinate (PBS), polylactic acid (PLA) and polybutylenes adipate-co-terephthalate (PBSA) have excellent biocompatibility, which guarantees the development of suitable biodegradable material [1,2,3]. Particularly because of polymer’s exceptional environmental friendliness, lightweight, high shock absorption, high thermal stability and low dielectric constant, the polymers show great promise for diverse applications, including insulation materials, automotive products, health sporting goods and packaging [4,5]. The PBS is made by mixing succinic acid and 1,4-butanediol and is sold commercially. Additionally, PBS is an aliphatic polyester with a lower glass transition temperature (Tg), high elongation at break and excellent toughness that is biodegradable [6,7]. PBS has been regarded as a potentially useful material for utilization in medicine, construction, and packaging due to its qualities of biodegradability, flexibility and toughness [8]. There is a growing interest in the study and advancement of electrospun PBS due to its exceptional compressive and impact properties as well as its improved viability and cost efficiency which can be extended to a variety of biodegradable PBS applications in broader domains [9]. Unfortunately, PBS’s application was limited due to its weak formability, which was caused by its relatively high crystallinity (χc) and poor melt strength [6,10]. To overcome these limitations, a variety of modification techniques have been attempted, including mixing and blending with other polymers, crosslinking, nanoparticle filling, chain extension and copolymerization [11,12,13,14,15]. Among these strategies, filling it with nanoparticles is an attractive way to increase its formability and give it multiple functions at once [16]. Because of the high-aspect ratio, low density and superior conductivity, multiwalled carbon nanotubes (MWCNTs) were frequently utilized in the creation of biodegradable polymer-based nanocomposites [17,18]. The influence of MWCNTs on the characteristics and properties of biodegradable nanocomposites has been documented in a number of studies [4,19,20]. In a prior study, electrospun PLA/CNTs nanofiber with an ultrahigh volume–expansion ratio was created using a straightforward, environmentally friendly method of 49.6 [4]. In addition, ternary composite of PBS/CNTs/PTFE was developed with excellent electrical conductivity (σ) by utilizing the process of ball milling and the carbon dioxide foaming process [21]. Adding CNTs to PBS generally reduces its biodegradability, mainly due to barrier effects, increased crystallinity and CNTs’ non-biodegradable nature. Functionalization or very low CNT loadings can reduce this negative impact, but overall, PBS/MWCNT nanocomposites are less biodegradable than neat PBS [22]. MWCNT was presented in few studies as it is integrated in biodegradable and elastic polymers as electromagnetic interference shielding (EIM) material [20,23,24,25]. The pressure-directed flow methodology was employed to develop an ultra-lightweight and high-strength carbon nanotube-reinforced polylactic acid nanocomposite with a noticeable compressive strength of 54 MPa, electrical conductivity of 3.4 S/m and superb specific electromagnetic interference shielding efficiency of 77 dB [24]. The fabrication of the freeze-drying process was used to create carbon nanotube reinforced polylactic acid nanocomposite with good EMI shielding properties and better resistance of heat [20]. Biodegradable electrospun nanocomposite with notable flexibility and elasticity was rarely reported, whereas some work concentrated on rigid and stiff biodegradable electrospun nanocomposite. Also, electrospinning promotes alignment and uniform distribution of MWCNTs within the polymer matrix, facilitating the formation of an effective percolation network even at low filler loadings. Electrospun fibers exhibit controlled porosity and fiber diameter, which can improve mechanical flexibility, thermal stability and potential electrical conductivity [25]. PBS is regarded as a great candidate material to create a lightweight, flexible electrospun nanocomposite since it is a biodegradable, flexible polymer. Because of these polymer properties, creating and designing an electrospun PBS/MWCNT composite for use in EMI shielding fields was a compelling research project [26,27,28]. Also, the electromagnetic interference (EMI) shield films made of thermoplastic polyurethane (TPU), polybutylene adipate-co-terephthalate (PBAT), 1-(2-aminoethyl)-3-methyl-1H-imidazol-3-ium modified MWCNT (MIL) and advanced rubber composites shows the EMI shielding effectiveness value from −17 to −24 dB [29,30,31,32,33].

In this paper, we present the development of an effective and eco-friendly biodegradable electrospun polymer PBS/MWCNT with EMI shielding characteristic to accomplish the intended results. The electrospin process of MWCNT dispersion in a PBS matrix, melting and crystallization behavior, mechanical property and electrical conductivity with the EMI shielding function of developed electrospun PBS/MWCNT nanocomposites were studied. Finally, the novel approach for creating environmentally friendly functional biodegradable materials with elasticity and flexibility and the biodegradable electrospun PBS/MWCNT nanocomposites with an EMI shielding characteristic also offer diverse commercial applications.

## 2. Materials and Methods

### 2.1. Materials

Showa Denko K.K. Japan supplied polybutylene succinate (PBS-3001MD). Multiwalled carbon nanotubes MWCNTs with greater than 96% purity and about 8–18 nm diameter were acquired from Nanografi Nano Technology, Ankara, Turkey. The solvents such as Chloroform (CHCl_3_-40021) and acetone (CH3COCH3-534064) were provided by SigmaAldrich, St. Louis, MI, USA.

### 2.2. Preparation of the Solution and Electrospinning Process

Five different types of PBS-based electrospun fibers were prepared including neat PBS and four concentrations of MWCNTs (0.5, 1, 2 and 3 wt%) in PBS matrix. Furthermore, PBS solution was developed by integrating a suitable amount of PBS in solvent mixture of chloroform/acetone having a ratio of 6:5 *v*/*v*. A two-step mixing procedure was used to create composite PBS solutions: first, a pure PBS solution was made, and then an equivalent quantity that matched the MWCNT concentration was added to the solution, as shown in Table 1. The solution was created in the ultrasonic bath for about 30 min for MWCNT dispersion in the PBS matrix. Also, after 24 h of mixing at room temperature with a magnetic stirrer, the prepared solution was relocated to a 5 mL syringe which is attached to the electrospinning machine TL BM (Shenzhen, China). The parameters of electrospinning process were accustomed for preparation of each specimen three times are shown in Table 1.

**Figure 1:**
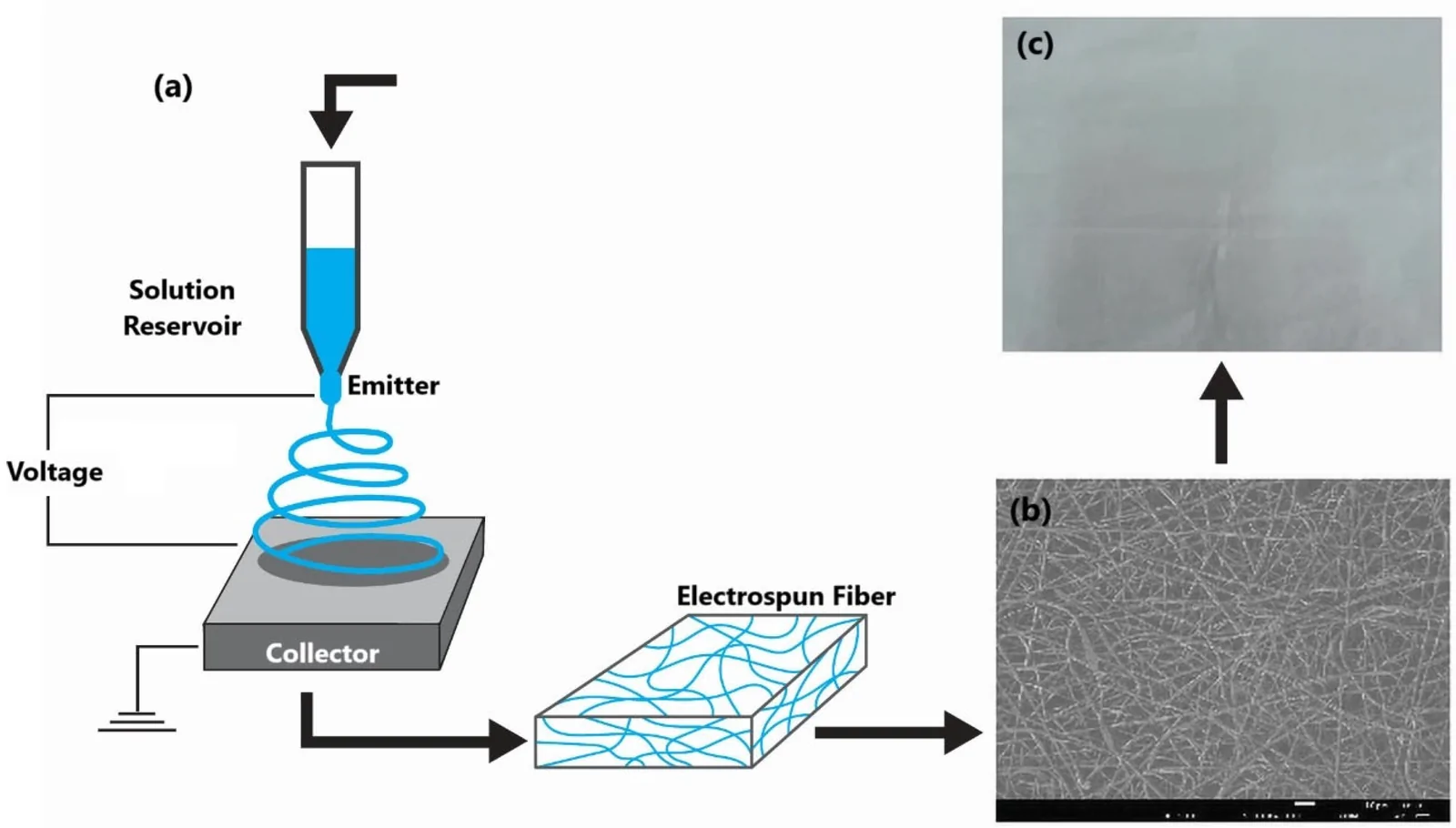
(**a**) Schematic representation of electrospinning process; (**b**) SEM image of electrospun fiber; (**c**) digital image of developed electrospun mat.

Finally, after electrospinning, all collected electrospun PBS/MWCNTs mats were detached from aluminum sheet and dried for 24 h in a vacuum oven at about 40 °C to remove any remaining solvent [5]. Figure 1 shows the electrospinning process with the SEM micrograph and digital image of the developed MWCNT/PBS electrospun mat.

### 2.3. Characterization

Morphological characteristics and elemental composition of electrospun fiber were studied by utilizing scanning electron microscopy (JEOL 7600F, Tokyo, Japan) with an energy-dispersive X-ray, EDX (Oxford Instruments, Tokyo, Japan) system. Also, a unique tailored processing image technique was used to estimate fiber size distribution.

The FTIR analysis of a PBS/MWCNT specimen was conducted by utilizing Thermo Scientific Nicolet 6700 FTIR (Thermo Fisher Scientific, Waltham, MA, USA). The spectrum was characterized in the 400–4000 cm^−1^ wavelength range.

With 633 nm excitation wavelength, Raman spectra was measured with the help of a LabRAM HR800 Raman spectometer (Horiba Jobin Yvon, Palaiseau, France).

The material was analyzed by implementing an X-ray diffractometer (XRD 7000 by Shimadzu Limited, Kyoto, Japan) by applying monochromatic Cu K radiation as 1.540598 nm. The two angles between the range of 10° and 50° were scanned with the scan speed of about 8.0 deg/min.

The thermal characteristics of various PBS/MWCNT specimens were then illustrated by utilizing a differential scanning calorimeter (DSC 200 F3, Netzch Bavaria, Germany). The process of heating and cooling was performed from 20 °C to 180 °C at 10 °C/min. Also, the acquired thermogram was used to inspect melting enthalpy, melting temperature, crystallinity Xc % and crystallization temperature. The crystallinity Xc % of electrospun composite was evaluated by using the following Equation (1):

(1) $$X_{c}\;\%=\;\frac{∆H_{m}}{wx\;∆H_{o}\;}\;\times \;100\;$$

where ΔH_m_ determines the melting enthalpy and ΔH^o^_m_ defines the enthalpy of neat PBS which is 110.3 J/g and w signifies PBS fraction of weight for PBS/MWCNT composite.

The Thermogravimetric analysis was conducted on SetaramSetsys Evolution-1750 instrument, Francheville, France. The specimen with average estimated weight of 5 mg was heated from 30 °C to 500 °C at 10 °C mm/min heating rate in an inert argon atmosphere. Additionally, during heating the specimen temperature and weight loss differences were noted as a function of temperature.

The drop shape analyzer (DSA 100, Bonn, Germany) was used to determine the wettability which was studied by static water drop on MWCNT/PBS composite. The captured water drop image on the electrospun composite surface was used to measure the contact angle by examining the profile of the drop with software. Also, the ultimate contact angle θ was acquired by using the mean of 5 measurements.

Mechanical properties of the electrospun MWCNT/PBS composite were concluded by employing a tensile testing machine, Instron 1122UK, with a 1mm/min crosshead speed. The specimens were simultaneously sliced from electrospun mats in the rectangular shape with approximate dimensions of 40 mm × 6 mm.

The loss factor (tan delta) and storage modulus G’ of MWCNT/PBS composites were studied by dynamic mechanical analyzer, DMA 242, Netzsch, Selb, Germany. The DMA machines operate with cantilever technique with the specimen size of 40 mm × 12 mm × 6 mm. In addition, temperature was maintained in between −60 °C and 80 °C. Furthermore, the tests were performed at a 1 Hz frequency, a heating rate of 2 °C/min and a strain rate of 0.1% in an N_2_ environment.

The electrical conductivity of the specimen was analyzed by using four-point probe equipment from Ossila, Sheffield, UK. The device is capable of creating voltages ranging from 100 μV to 10 V and also currents in the range of 10 nA–100 mA. The thickness of specimen differed from 0.3 to 0.4 mm, and sheet resistance was calculated at three discrete positions. The conductivity was calculated by using the following equation [29]:

(2) $$\sigma =\;\frac{1}{R_{s}\;t}$$

In the equation, σ is the electrical conductivity (S/m), Rs (Ω/Sq) is the sheet resistance and t is the sample thickness (m).

In addition, the AC impedance analysis of the PBS/MWCNT composite was carried out by AC impedance analyzer in between the range from 0 to 10^7^ Hz with an approximate potential difference of about 1 V. Also, the complex AC impedance (Z) deals with two components: a real part (Z’) which indicates resistivity and an imaginary part (Z’’) which provides reactance experienced by the AC field due to the presence of polymer nanocomposite. The data from the AC impedance analysis are utilized to calculate AC conductivity with the help of Equation (3) [30].

(3) $$\sigma_{c}=\left(\frac{e}{s}\right)\;\left[\frac{Z^{\prime }}{{{(Z}^{\prime }}^{2}+{\;Z^{\prime \prime }}^{2})\;}\right]$$

In the equation, e is the thickness and s is the area of contact found in the polymer matrix in proximity to the electrode plate.

The EMI shielding characteristic of PBS composite was studied by using a vector network analyzer, VNA, Agilent E5071 C, Santa Clara, CA, USA, with a waveguide way in the X band range of 8.2–12.4 GHz. Also, standard PBS/MWCNT specimens were developed and the dimensions of 14 mm × 2 mm were placed in a holder of the waveguide for measurement.

## 3. Results and Discussion

### 3.1. Morphology of Fibers

**Figure 2:**
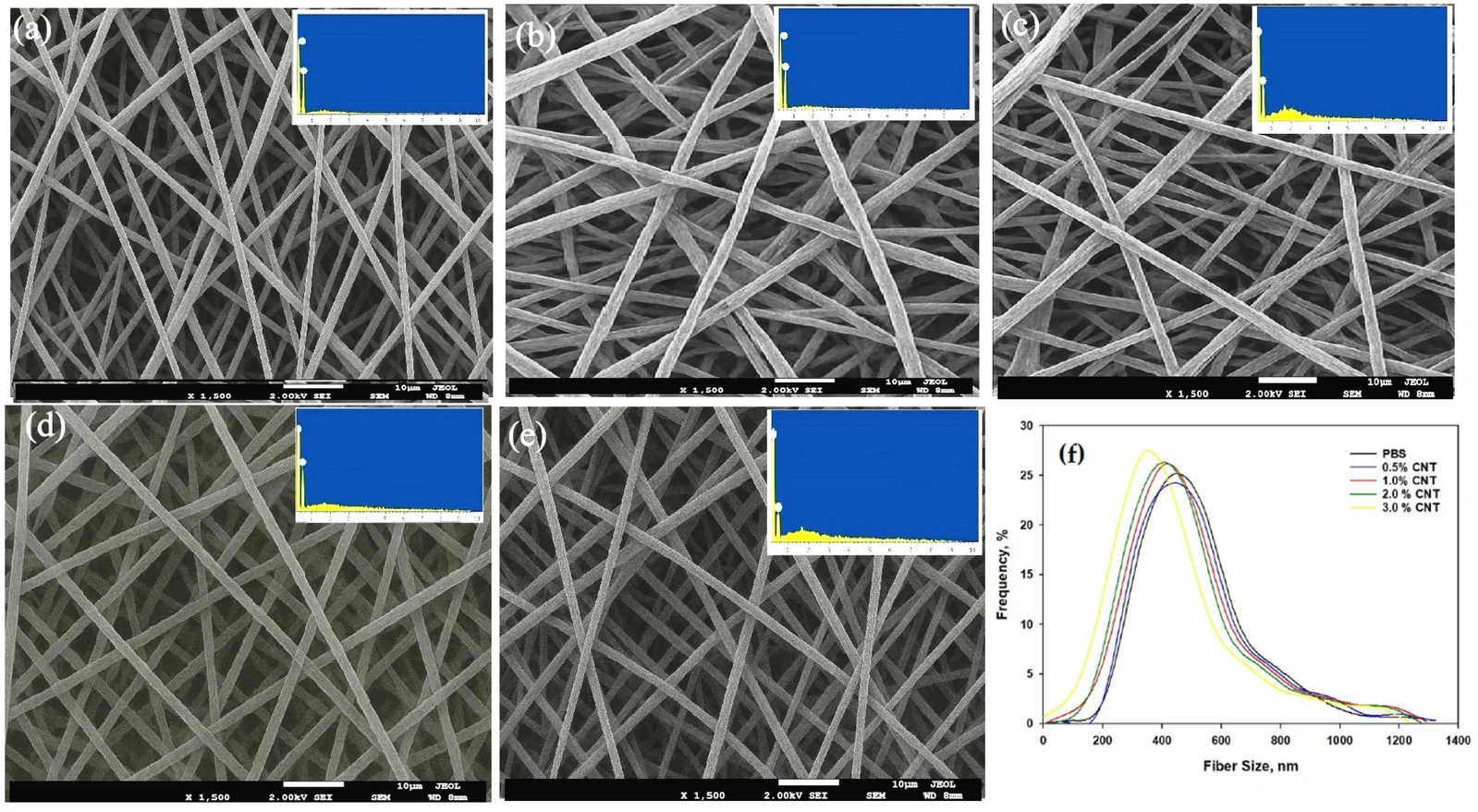
SEM of electrospun MWCNT/PBS fibers: (**a**) PBS, (**b**) 0.5% MWCNT/PBS, (**c**) 1.0% MWCNT/PBS, (**d**) 2.0% MWCNT/PBS, (**e**) 3.0% MWCNT/PBS, (**f**) MWCNT/PBS Fiber size.

Figure 2 presents SEM photographs of neat PBS and MWCNT/PBS electrospun nanofibers. It can be noticed that uniform and smooth fibers are obtained without any beads because electrospun drops were fabricated by employing appropriate procedure parameters including needle-to-collector distance, feed rate and voltage, as presented in Table 1. The inclusion of MWCNT does not disturb the morphological structure of nanofibers by considering the parameters. The beadless and smooth fibers were acquired with the implementation of prepared PBS-based solution which contained MWCNTs (Figure 2b–e). During the early formation of the fibers, the solvent that is trapped between and around MWCNT entirely evaporates and leaves behind a homogeneous and noticeable smooth surface. Figure 2f demonstrates the size distribution of fibers. Also, 3.0 wt. % MWCNT fiber shows a diameter of 365 ±nm which is about 25% smaller than neat PBS fiber which is around 450 nm. Similarly, the 0.5, 1.0 and 2.0 wt.% MWCNTs are around 435 nm, 410 nm and 390 nm. Since the electric field has an impact on solution, the properties of solutions control the overall morphology of fiber, including its size. The phenomenon may arise from the presence of pure PBS during the fiber’s flight time, which creates a resistivity potential that prevents fiber from thinning in an electric field. Furthermore, because of the presence of higher conductivity of PBS polymer solution with the addition of MWCNTs, the elongation of viscoelastic mixture in high-voltage electric fields is significantly increased which results in a lower diameter of 3% MWCNT fibers [22].

The EDS spectrum image of pure polybutylene succinate (PBS) notably shows sharp peaks for carbon and oxygen which are main components of the polymer (C_8_H_12_O_4_)n. The EDS did not detect any presence of hydrogen, although it was present. Moreover, carbon contributed to a majority of weight and the atomic percentage as it is a main structural component. Meanwhile, the peaks for oxygen appeared from the presence of carbonyl and ester groups. The addition of MWCNT in PBS makes the peaks for carbon climb with a lowering of peaks for oxygen as shown in Table 2.

An optimized PBS concentration of 6 wt.% was employed for electrospinning, as it provided the requisite balance between solution viscosity and fiber formation while eliminating bead defects. The chloroform and acetone as 3:1 *v*/*v* solvent system was selected as chloroform successfully dissolves PBS, warranting homogeneous solution. Meanwhile, the application of acetone decreases the viscosity of solution and also increases volatility, which results in the formation of smooth fiber during the process of electrospinning. This particular combination forms uniform shapes and free of bead nanofibers with regular a diameter which as a result improves the dispersion of MWCNT and overall performance of composite.

### 3.2. Functional Group Analysis

**Figure 3:**
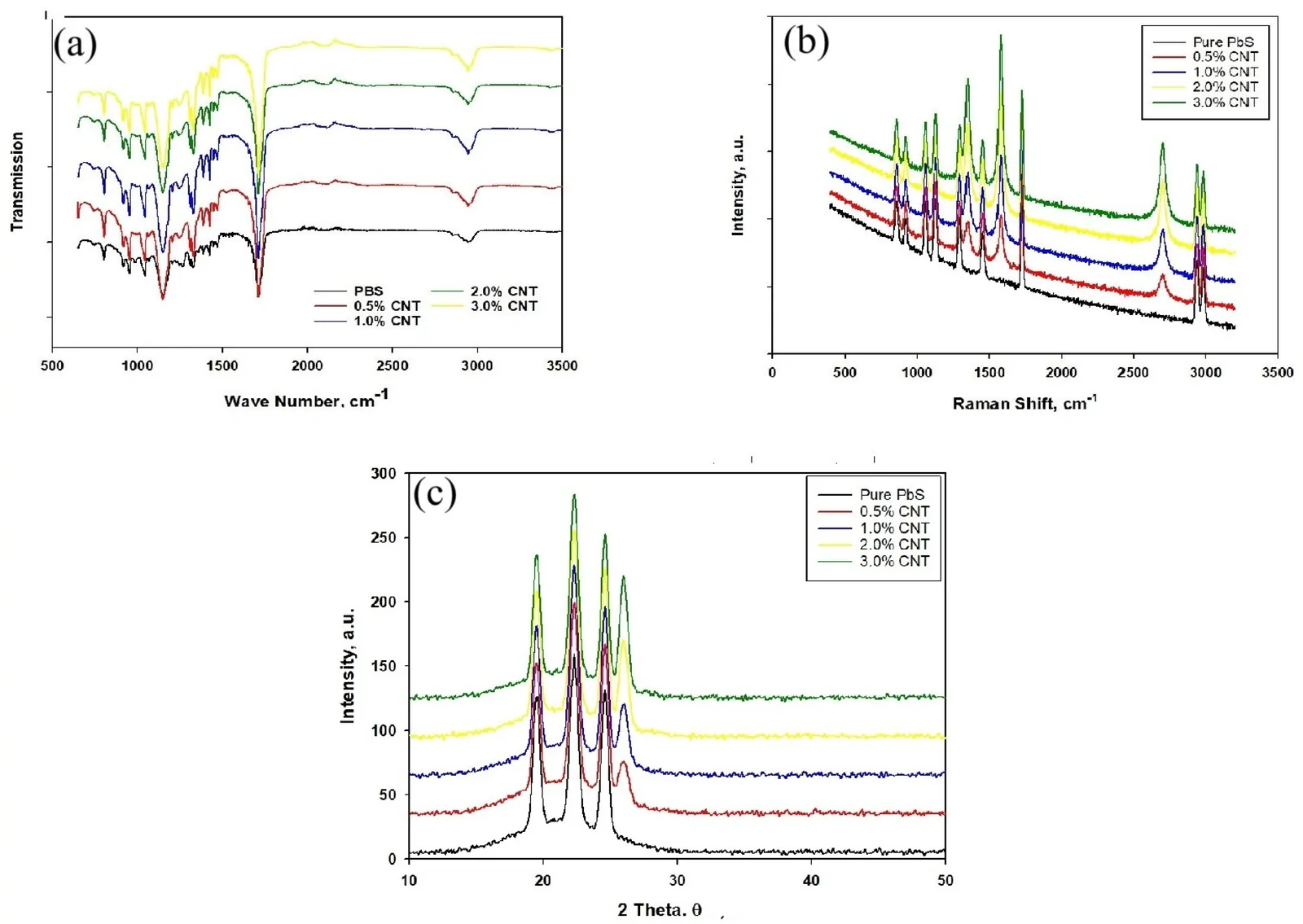
MWCNT/PBS electrospun fiber: (**a**) FTIR spectrum, (**b**) Raman spectra, (**c**) X-ray diffraction pattern.

The functional group of the electrospun PBS/MWCNT nanocomposite is being presented in Figure 3. The characteristic peaks of PBS are shown as follows: 2940—CH_3_, 1732—C=O, 1675—CH_2_, 1379—CH_3_, 1437—CH_3_. Also, hydroxyl has its presence in the PBS polymer structures. A similar pattern was also noticed when MWCNT is mixed in PBS solution. The repetitive form of –OH was distinctly exposed due to hygroscopic character of the MWCNT. Also, inclusion of the MWCNT in PBS added attributed peaks at about 1066 cm^−1^ and 1166 cm^−1^ for C–O, 1599 and 1447 cm^−1^ for the aromatic ring and 1748 cm^−1^ for C=O. Additionally, peaks are formed at about 1750 and 1061 cm^−1^ for MWCNTs because of the existence of stretching vibrations C=O, and carboxyl group C–O [19]. In addition, the solution demonstrated an effective dispersion of most MWCNTs in solvent such as chloroform and also reveals the tendency of PBS adhesion characteristics. Also, the addition of 0.5 % MWCNT in PBS moved the peak bands from 1748 cm^−1^ to 1743 cm^−1^ and 2996 cm^−1^ to 2990 cm^−1^. This phenomenon occurred due to structural changes because of a large number of functional groups [19]. The identical behavior is noticed with all the concentrations of PBS composite. A clear fascinated peak can be noted at 1749 cm^−1^ related to stretching of C=O, illustrating the creation of strong van der Waals forces due to the occurrence of considerable adhesion and lattice properties of the PBS which might have led to a considerable affinity between MWCNT and PBS [19].

The Raman spectrum for pure polybutylene succinate PBS demonstrated noticeable vibrational peaks around at about 860–1730 cm^−1^ which corresponds to C–O, C–C and ester C=O in stretching modes [28]. Also, incorporation of carbon nanotubes (CNTs) into PBS shows supplementary characteristic bands including the D band ~1350 cm^−1^ corresponding to the SP^2^ carbon ring, G band ~1580 cm^−1^, sp^2^-hybridized carbon and the 2D band ~2700 cm^−1^ relating to the carbon ring which have a special signature presence of graphitic natured carbon structures. Also, the coexistence of PBS molecular vibrations with the CNT bands verifies excellent reinforcement of the polymer matrix with implemented CNTs [28]. The newly formed functional groups improve dispersion within the PBS matrix and augment interfacial interactions by hydrogen bonding as well as dipolar interactions finally impacting both hydrophobicity and mechanical performance of developed composites.

The XRD pattern of PbS integrated within CNTs predictably displays characteristic cubic rock salt structure for PbS with outstanding diffraction peaks indexed to 111, 200, 220, 311 and 222 planes at about 2θ ≈ 25.9°, 30.0°, 43.0°, 51.0° and 53.4°. Zhang etc. [17] also found similar results. The extensive background from CNTs might overlap with several PbS reflections particularly at around 26° where CNT 002 peak appears representing probable interfacial interactions or a partial encapsulation of PBS in the CNT configuration.

### 3.3. Thermal Characteristic

**Figure 4:**
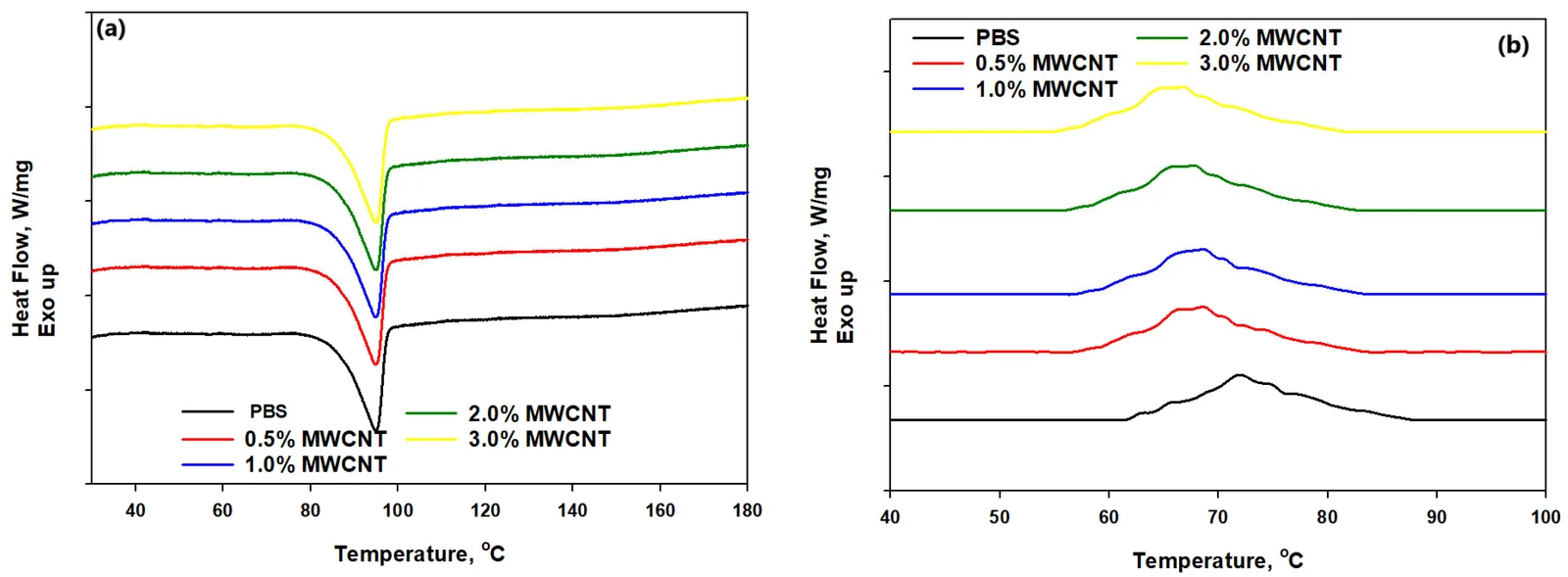
DSC thermogram of MWCNT/PBS fiber: (**a**) heating, (**b**) cooling.

Figure 4 demonstrates melting and cooling curves of the PBS with different contents of MWCNT. The obtained thermal characteristics and properties are presented in Table 3. Figure 4a and Table 3 demonstrate that the melting temperature (Tm) of neat PBS is about 98.6 °C and the inclusion of MWCNTs enhances a clear shift of the Tm to a higher temperature. Meanwhile, when the CNT concentration becomes 3 wt%, the Tm of nanocomposite increases by about 103.2 °C which is 4 °C higher than the neat PBS and which also slightly increases melting enthalpy. The behavior occurs because of MWCNTs’ heterogeneous nucleation effect as it reduces the energy barrier for molecular PBS chains to develop crystal nuclei [30,31].

Figure 4b shows DSC cooling curves of different PBS samples as it shows cooling peaks which could be explained by the recrystallization progression of PBS in a cooling process [34]. The Tc of PBS samples is noticed to be around 78.8 °C. The 3% rise in MWCNTs content resulted in a 5 °C increase in Tc cooling temperature and the cooling enthalpy in comparison to neat PBS as described in Table 3. The behavior suggests that MWCNTs played an important function in the formation of strong PBS crystallites with a stable structure [35]. Moreover, Figure 4b shows there is a slight increase in crystallization of PBS samples when the MWCNT content is increased. The increase may have occurred because of two reasons: firstly, the addition of MWCNTs as an effective heterogeneous nucleation site which could have facilitated the process of PBS crystallization nucleation [32]; secondly, entanglement in between the PBS molecular chains and the CNTs as well as the development of MWCNTs networks hindered molecular PBS chain movement which as a result limited growth of PBS spherulites. From a practical standpoint, although the magnitude of the Tc and Tm increase is not dramatic, it does have processing implications. A higher Tc indicates that PBS/MWCNT composites solidify more readily during cooling, potentially enabling faster cycle times in melt-processing operations such as injection molding or extrusion. Likewise, the slight increase in Tm marginally broadens the processing window by allowing thermal shaping at slightly higher temperatures without premature softening. In terms of application performance, the raised Tm may translate to improved dimensional stability and service temperature, especially under conditions close to the softening point of neat PBS. Thus, even modest improvements in Tc and Tm reflect the role of CNTs as effective nucleating agents, with positive implications for both manufacturing efficiency and thermal performance of the composites.

**Figure 5:**
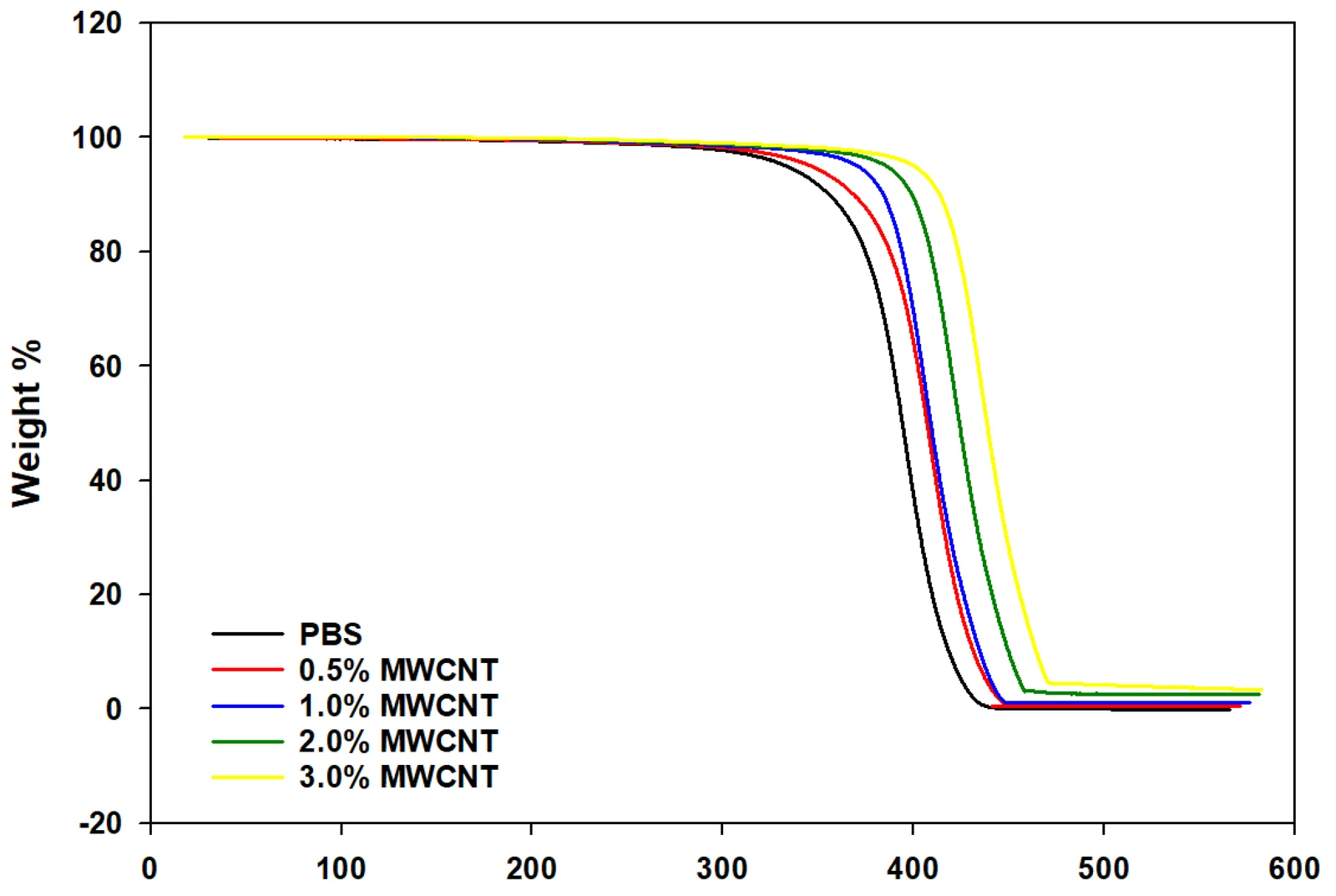
TGA of MWCNT/PBS fiber.

The outcomes of thermogravimetric scans are described in the Figure 5 which shows weight loss of about 0% in range of 30–220 °C for the neat PBS. The degeneration of neat PBS happens in range of about 229 °C and 368 °C showing loss of weight around 99.17% due to thermal decomposition of the neat PBS hydrocarbon molecular chains. The 15–25% weight loss was observed when 0.5–3.0% MWCNT was included in the PBS composite. The weight loss of about 72–77% occurred in between 278 °C and 374 °C when almost 0.5–3.0% MWCNT was included [33]. The study shows that thermal stability of MWCNT as thermal energy is degenerated equally which can be due to the presence of Van der Waals forces in electrospun fiber [34]. The obtained results demonstrate that complete degradation of PBS at 455 °C can be prohibited when MWCNT is slowly increased as it is related to ash. The improvement in the thermal stability might be due to the well distribution and dispersion of MWCNT in the PBS matrix which leads to act as an efficient hindrance effect to the volatile compound developed during PBS decomposition.

Additionally, to the beginning and maximum degradation temperatures, the residual mass at the end of TGA flow should also be contemplated, especially for MWCNT incorporated composite. Also, neat PBS decays virtually fully and leaves insignificant residue, whereas the PBS/MWCNT composites demonstrated more residual weight that rises with MCNT content. The residue begins from inherited thermal stability of MCNT fibers and the presence of catalyst resultant inorganic impurities. The higher char yield studied in composites confirms efficient incorporation off MWCNT and shows a relationship with their concentration. The final residues are vital not only as the indirect measure of CNT loading, but also because the residue may affect the electrical and thermal performance of final composite.

### 3.4. Mechanical Properties

**Figure 6:**
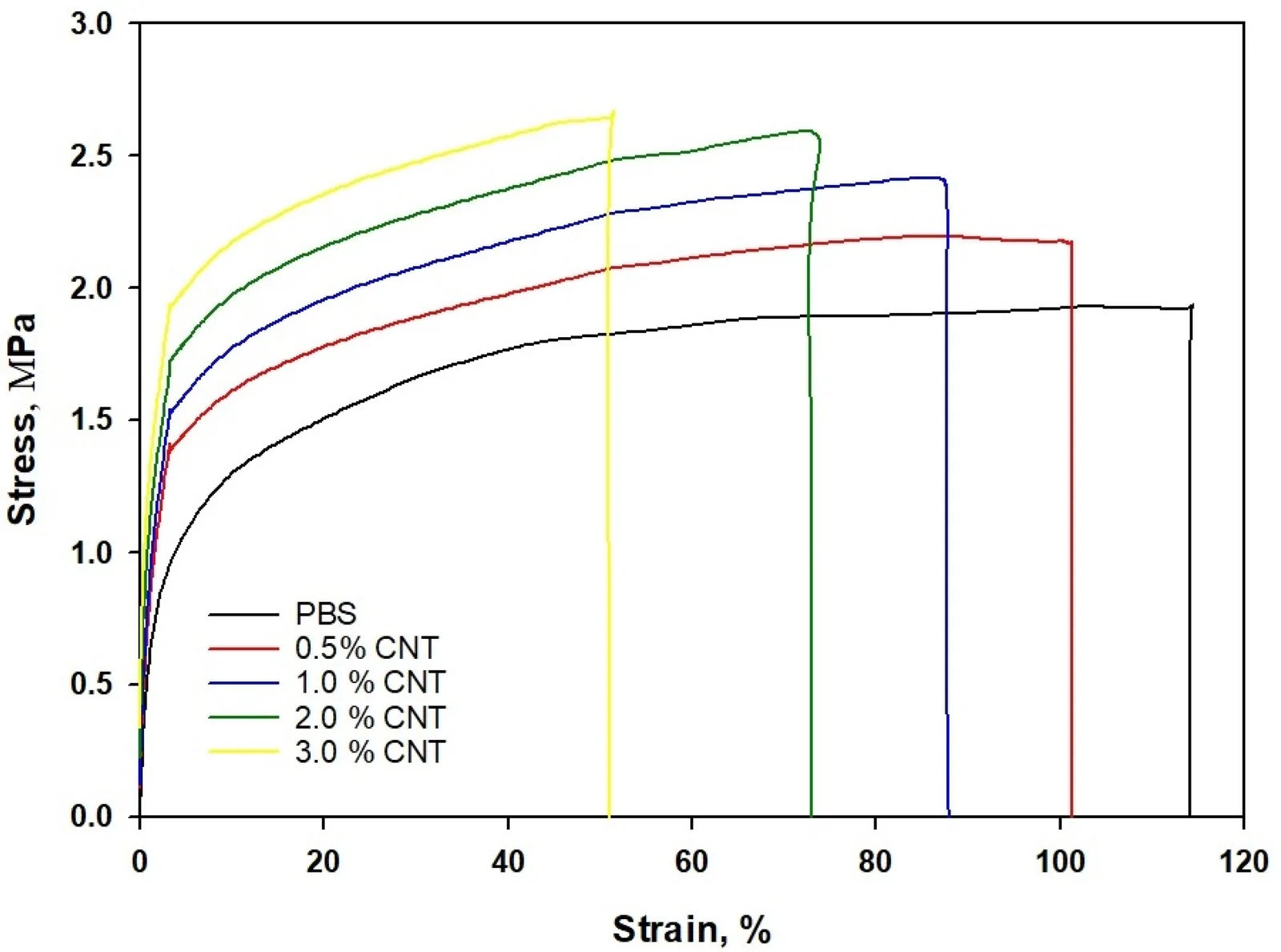
Tensile curve of MWCNT/PBS electrospun mat.

Figure 6 shows the typical stress strain behavior for MWCNT/PBS electrospun fiber. Table 4 shows tensile strength of a MWCNT/PBS electrospun mat with different concentrations of MWCNTs. It is noticeable that strength of the PBS is 1.81 ± 0.17 MPa. Also, the strength of PBS-3.0 wt.% MWCT composites increases to 2.61 ± 0.15 MPa when content of MWCNT is increased inside the PBS matrix. At the same time the modulus of elasticity also increased to 0.72 GPa with the addition of MWCNT. Table 3 demonstrated that there is a small change happened in crystallinity of PBS with the inclusion of MWCNT signifying that crystallinity insignificantly influences the strength of electrospun PBS/MWCNT composites [34].

The final results demonstrated that enhancement in the elastic modulus and mechanical strength of electrospun PBS/MWCNT composite occurred because of adequate dispersion and presence of interfacial bonds between the PBS and MWCNT. Furthermore, enhanced tensile strength illustrates that force is equally and uniformly relocated from the PBS matrix to MWCNT. Also, Table 4 shows that % elongation at the break of PBS due to increase in the content of MWCNT. The neat PBS has about 99.15% elongation which decreased to 75% when the quantity of MWCNT in PBS matrix is increased. Also, a 24.5% reduction in the MWCNT/PBS nanocomposite shows the decrease in ductility because of dispersion and noticeable interaction between the MWCNT and PBS matrix [36]. Moreover, % elongation at the break lowers as content of the MWCNT increases that is stiff in nature and acts as a hindrance for polymer chain mobility but also still is flexible. As various inconsistency is inherent with the process of electrospinning, the process was found to be highly replicable under controlled states and conditions with some batch-to-batch slight deviations within ±0.3 range for the tensile strength and ±0.5 for the elongation at break. This consistency highlights the reliability of the process of electrospinning as a scalable production technique. Meanwhile, it also ensures that examined mechanical improvements originate from reinforcing influence of MWCNTs rather than fabrication artifacts.

**Figure 7:**
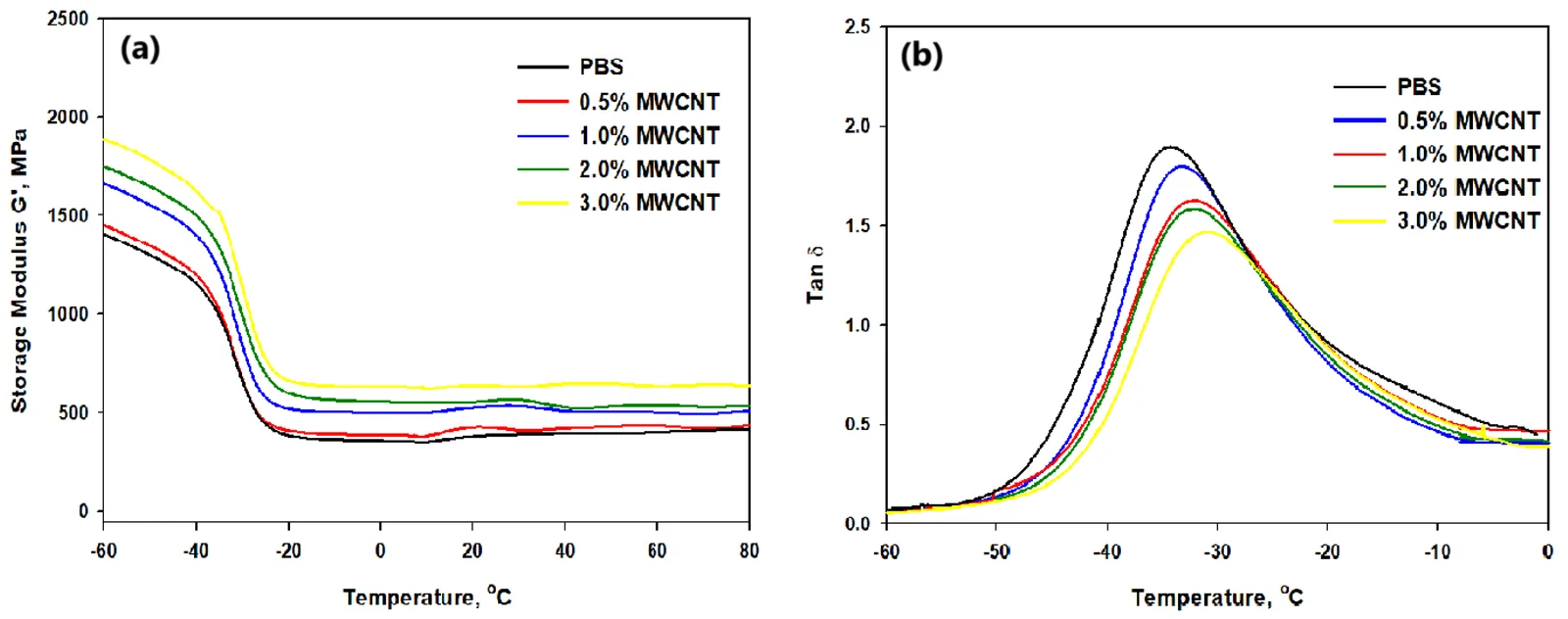
Dynamic mechanical analysis: (**a**) storage modulus, G’; (**b**) Tanδ.

Figure 7a shows storage modulus G’ of PBS/MWCNT composite with the function of temperature. The G’ of neat PBS is 1667 MPa and it increases to 2038 MPa when MWCNT is added. The rise of G’ for the PBS/MWCNT specimen occurs due to interfacial reaction and strong adhesion between MWCNT and the PBS matrix. Also, the 21% increase in storage modulus happens because of enhancement in the stiffness of the PBS matrix as MWCNT adds effects of reinforcement [37,38,39]. The glass transition temperature (Tg) resulted in a rapid fall at -32 °C of storage modulus G’ related to all specimens. The fall in storage modulus G’ can be justified by PBS molecular chain mobility which becomes above Tg. The contrast between the rubbery and glassy state of the studied storage modulus demonstrates the reinforcement effect visible in electrospun PBS and PBS/MWCNT composite.

Figure 7b demonstrates that the loss tangent, tan δ, of PBS is greater in comparison to PBS/MWCNT composite. This is evident that the peak height value of neat PBS is 1.9 when compared to 0.5% MWCNT/PBS composite with a value of 1.72 and 3% NFC/MWCNT had a value of 1.46. The height of tan δ decreases with the increasing concentration of MWCNT which defines that PBS/MWCNT composites become rigid [40]. Also, molecular relaxation increases because of a decline in tan δ which helps in the large absorption of energy in the PBS/MWCNT composite. Furthermore, significant molecular relaxation and excellent absorption of considerable energy is gained for a 3%MWCNT presence in the PBS. The occurrence of robust interfacial bonding and adhesion increases noticeable absorption activities which help in lowering energy loss and dissipation in the PBS/MWCNT composite [35]. The incorporation of MWCNT in PBS lowers the tan δ enhancing elastic attribute and decreases the energy dissipation for MWCNT/PBS composites when compared to the PBS specimen as shown in Table 4. Also, Figure 7b describes tan δ peak for evaluation of glass transition temperature. Also, it is evident that the Tg significantly increased to −30 °C for 3% MWCNT/PBS composite when contrasted with neat PBS which has Tg of −35 °C. The change in Tg may have occurred due to obstruction in chain mobility affecting realignment of segments during phase transition [41,42].

### 3.5. Wetting Behavior

**Figure 8:**
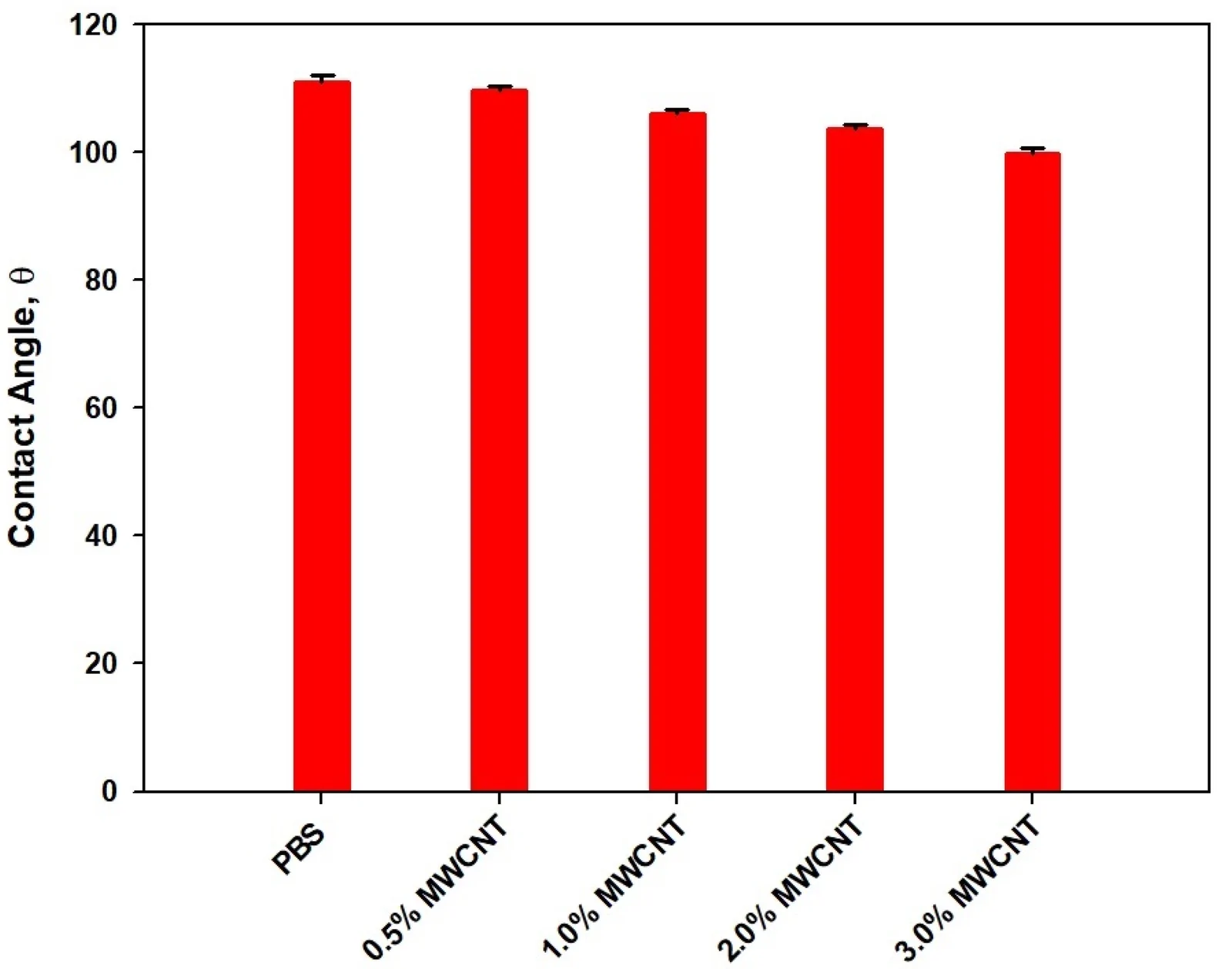
Wetting behavior of MWCNT/PBS.

Figure 8 shows wetting behavior with respect to contact angle of PBS and the MWCNT/PBS composite. The contact angle for neat PBS appears to be 110.1° corresponding to hydrophobic nature. The hydrophobicity of PBS may be because of crystalline affinity which is assisted by results from DSC. (The hydrophobicity showed by PBS occurred may be because of crystalline affinity which is assisted by results from DSC.) The inclusion of MWCNT to PBS changed physical behavior and characteristic of MWCNT/PBS composite rendering it as hydrophilic. The 3.0 wt. % MWCNT has contact angle of 99.7° which is 9% lower when compared to neat PBS. The functional group like hydroxyl and carboxyl related to the wall and surface area of carbon nanotube affects the hydrophobic ability of PBS [19]. The hydrophobic nature conserve EMI shielding property and also adds qualities such as self cleaning, waterproofing and antibacterial capability.

### 3.6. Electrical Conductivity

The ability of electrical conductivity of any polymer nanocomposite relies on morphology. The morphology of polymer matrix shows confinement related to conductive nanoparticles inside the polymeric phase with loading data of conductive nanoparticles in the PBS matrix. Also, the diameter of conductive MWCNT performs a significant part in assessing the electrical conductivity of the resulting polymer nanocomposite at a certain nanofiller loading percentage.

**Figure 9:**
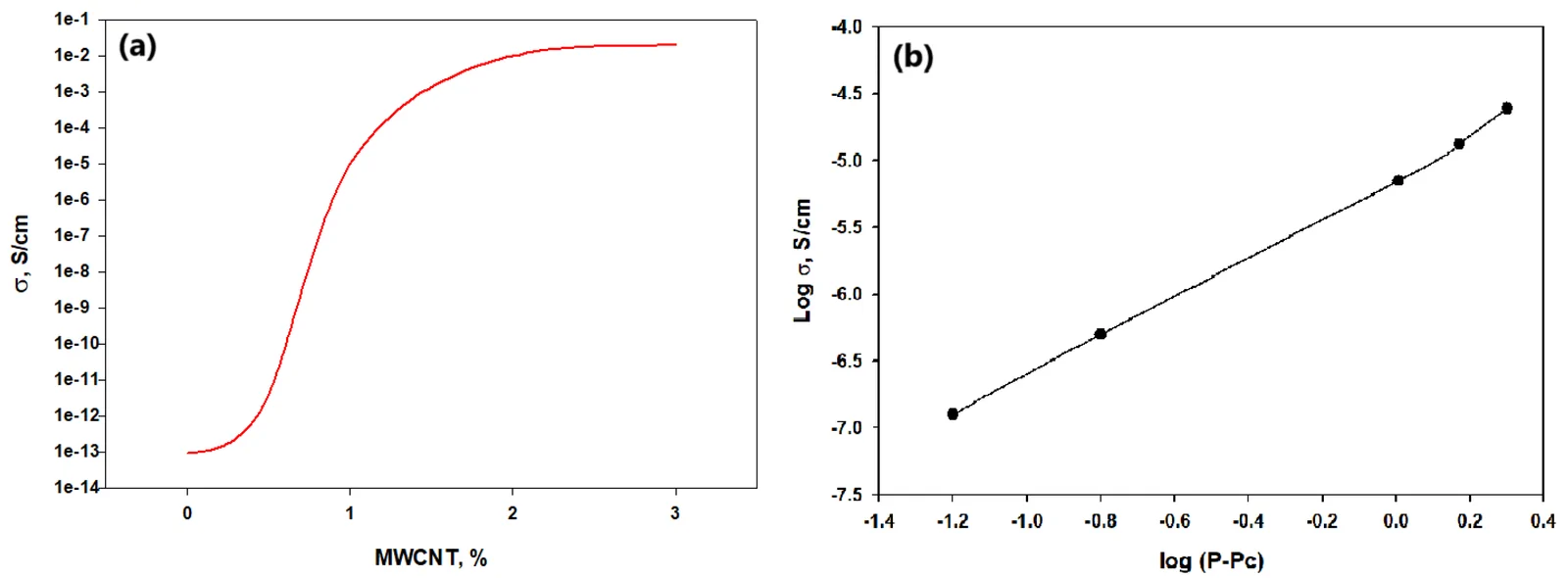
(**a**) Electrical conductivity, σ, of MWCNT/PBS nanocomposite; (**b**) fitting lines of electrospun MWCNT/PBS nanocomposite by percolation threshold method.

Figure 9a shows reliance of σ on MWCNTs content on the PBS. It is evident that neat PBS had a low σ of 7.8 × 10^−14^ S/cm and acted as an insulator [43]. Also, increasing MWCNT content implemented a steady increase in conductivity. The increase of 9 orders of magnitude is shown in Figure 9a for σ of around 1.9 × 10^−1^ which had a 2% MWCNT content. The results show a percolation threshold performance of MWCNT network in PBS/MWCNT nanocomposites [44]. The high MWCNT loading of 3% increases the conductivity, σ, of PBS/MWCNT nanocomposite which is not diverse anymore in comparison to 2% MWCNT. The increase in conductivity (σ) of PBS/MWCNTs electrospun nanocomposites can be attributed to improvement in EMI SE [37].

The percolation threshold of MWCNT/PBS electrospun nanocomposite was calculated by the statistical and mathematical percolation model which is shown as Equation (4) [38]

(4) $$\sigma =\sigma_{o}\;{\left(P-{P\;}_{c}\right)}^{t}$$

where σ is the conductivity, P represents MWCNT content in the PBS specimen, σ_0_ is relative constant deals with MWCNT, P_c_ is electrical percolation threshold and t is typical exponent related to conductive magnitude of PBS/MWCNTs electrospun nanocomposite. Figure 9b shows the linear affiliation of log σ when compared to log (P − P_c_). Also, percolation threshold capability of PBS/MWCNTs electrospun nanocomposites which is Pc = 0.94 wt% was evaluated and can be related to the presence of significant entanglement of the MWCNTs and MWCNT network in the PBS matrix as a result leads to the creation of conductive pathways [40]. Furthermore, with respect to PBS/MWCNT electrospun nanocomposite, ‘t’ is equal to 1.75 ± 0.04, describing wide and extensive tunneling distance allocation and the presence of a three dimensional conductivity system and network [39,40,45,46].

**Figure 10:**
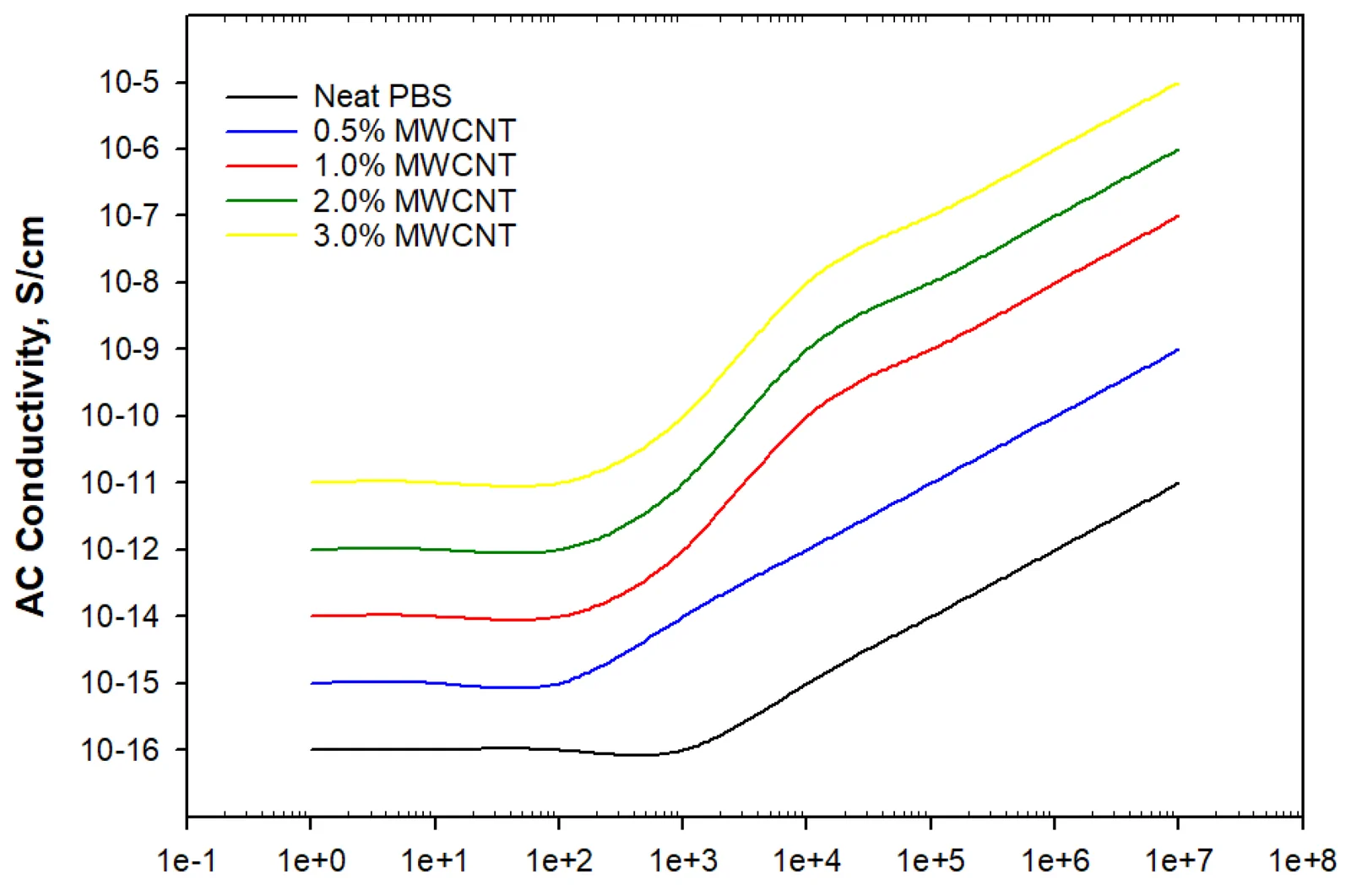
AC conductivity of electrospun MWCNT/PBS nanocomposite.

The PBS nanocomposite material with low MWCNT content demonstrates higher frequency dependency on AC conductivity in Figure 10. The PBS nanocomposite with a lower filler concentration of MWCNT appears to be close to each other with an increase in the frequency and the mobile charge transporter on PBS surface also comes closer which supports better electrical conductivity. Moreover, beyond the electrical percolation threshold, MWCNTs come close to each other and AC conductivity cannot be affected by increasing frequency. Also, PBS polymer nanocomposite with a high MWCNT concentration follows as a particular frequency known as the threshold frequency. After passing through the threshold frequency, the AC conductivity again becomes frequency reliant. Nath et al. [30] has reported the analogous performance when multiwalled carbon nanotube blended into the poly (butylene adipate -co-terephthalate) phase of the polymer matrix. When the filler concentration is higher, the interconnected network affects electronic transitions within MWCNT particles. The resulting consequence demonstrates that 3 wt%. MWCNT-reinforced PBS nanocomposite illustrates less frequency-dependent behavior with respect to lower MWCNT concentrations. Also, at the threshold frequency, the mobile charge carrier on MWCNT nanoparticle surfaces comes under the control of the AC field and starts to become aligned with the field. In addition, the threshold frequency for PBS nanocomposites with 0.5, 1, 2 and 3 wt.% of MWCNT concentrations are approximately 10,000, 80,000, 150,000 and 240,000 Hz.

### 3.7. EMI Shielding

The EMI SE is the critical logarithmic ratio of the incoming, P_in_, to outgoing, P_out_, power which is shown as dB. The total EMI shielding, SE_T_, shielding of absorption, SE_A_, and shielding of reflection, SE_R_, were achieved by recording most reflection coefficient (S11) parameters and transmission coefficient (S21) parameters. The EMI SE_T_ is calculated by using Equation (5) [47]:

(5) $${EMI\;SE}_{T}=\;{SE}_{R\;\;}+\;{SE}_{A}=10\log\left(\frac{P_{in}}{P_{out}}\right)\left(db\right)\;$$

**Figure 11:**
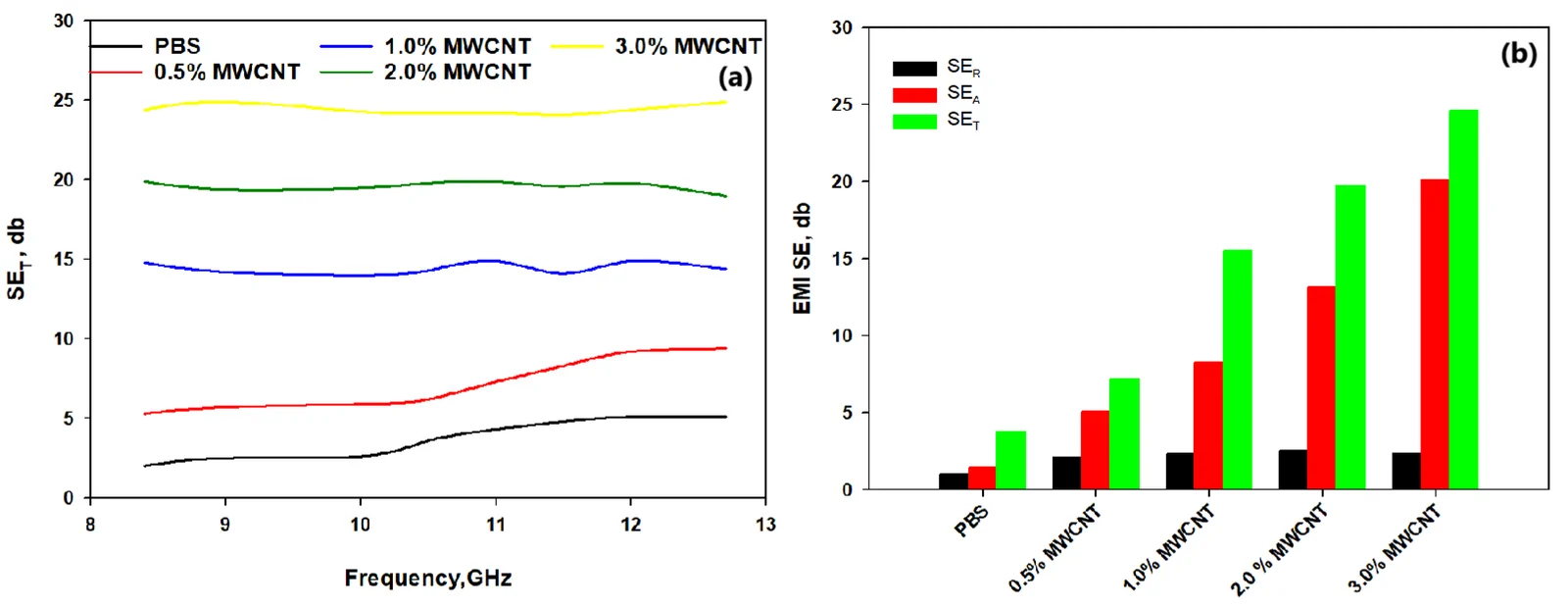
(**a**) SE_T_ of electrospun MWCNT/PBS nanocomposite with respect to frequency; (**b**) average values of SE_T_, SE_R_ and SE_A_ of electrospun MWCNT/PBS nanocomposite.

The EMI shielding properties of variable concentration of PBS electrospun nanocomposites with 1 mm thickness at X-band of 8.2 to 12.4 GHz were studied. Figure 11a shows SE_T_ for MWCNT/PBS specimens across a complete frequency range of X-band. Furthermore, Figure 11a demonstrated that the neat PBS showed less value of SE_T_ which can increase significantly by the presence of MWCNT dispersion [48]. The high SE_T_ of 25.5 dB was observed when MWCNT loading was about 3 wt% which exceeded the target level of 20 dB for beneficial industrial commercial applications [49,50,51]. Improvement of SE_T_ levels can also be related to the creation of MWCNT networks and structural arrangement in the PBS matrix which potentially can tolerate an increased amount of radiation of EM waves [52].

The mechanism of shielding of PBS/MWCNTs nanocomposites can be explained by differentiating the influence of SE_R_ and SE_A_ when they are detached from the SE_T_. Figure 11b illustrates average values SE_R_, SE_A_, and SE_T_ for MWCNT/PBS nanocomposites. It was noticed that the SE_A_ demonstrated a comparable pattern like SE_T_ and it also contributed mostly to SE_T_ which defines that the absorption was an influential mechanism. The average EMI SE_T_ value of 2.0% PBS/MWCNTs of nanocomposite is about 20.25 dB which can also be successfully used in commercial application as the requirement is 20 dB [53]. Meanwhile, an average EMI SE_T_ value of 1.0% PBS/MWCNT nanocomposite is only about 14.28 dB. Furthermore, the study illuminates that the developed electrospun PBS/MWCNTs nanocomposites demonstrate an excellent EMI shielding capability of 27.5 db even when a lower amount of MWCNT as filler was incorporated. Finally, when compared to PBS/MWCNT nanocomposites, the superb EMI shielding properties of 3.0% MWCNT nanocomposites can be credited to dense conductive network developed by virtue of the presence of its segregated structure.

The electrospun architecture produces highly porous, interconnected fiber networks that enhance multiple scattering and absorption, suggesting strong potential for absorption-dominated EMI shielding. The EMI shielding of electrospun PBS/CNT is 27.5 dB in the X-band which is better than bulk PBS/CNT films of similar filler content which is around 25 [22]. Thermodynamically, electrospun fibers generally exhibit lower Tg, Tm and TC compared to bulk counterparts because of their higher surface area, rapid solidification and polymer–nanotube interactions that disturb crystallinity. Also, MWCNTs act as nucleating agents typically dependent on small shifts of Tc and Tg upward by a few degrees and produce small increases in Tm [20]. Thus, electrospun PBS/CNT fibers are expected to trade some thermal stability for lighter weight, higher surface activity and potentially superior specific EMI shielding effectiveness.

The absorption-controlled shielding phenomena is also being witnessed for some carbon-based polymer composite. Also, SE_A_ is the function of conductivity, frequency of the incident wave and measured material thickness. The process shows that when material thickness is increased, the EMI SE values of composites demonstrate monotonic increments. The modification in shielding efficiency with the electrical conductivity, depth and material thickness cannot be ignored when developing superior quality EMI shielding material. Finally, electrospinning enables the production of PBS/MWCNT composites with optimized structural, electrical and mechanical performance.

The addition of about 3 wt% MWCNTs can considerably increase material costs due to the lofty price of carbon nanotubes. In addition, to analyze market competitiveness, we estimated cost per unit area of EMI shielding effectiveness (SE). By assuming a composite thickness of t mm, area A m^2^ and measured EMI SE of X dB, the cost per dB per m^2^ can be expressed as

$$Cost\;per\;dB.m^{2}=\frac{Materials\;cost\;per\;m^{2}}{EMI\;SE\;(dB)}\;$$

The addition of 3 wt% MWCNT increases the PBS cost from 8.28 USD/m^2^ to 14.34 USD/m^2^, but the shielding efficiency rises radically to 27 dB, dropping the cost per unit of shielding from 11.82 USD/dB·m^2^ for pure PBS to 0.48 USD/dB·m^2^. The mentioned analysis shows that as MWCNTs increase material cost, the shielding efficiency gained per dollar spent is far superior, stressing the advantage of incorporating CNTs.

## 4. Conclusions

The preparation of biodegradable electrospun PBS/MWCNTs composite with superior EM shielding characteristics is designed as an adjustable, cost effective and ecologically friendly process. The SEM micrographs present the development of a MWCNT/PBS fiber-based structure without the presence of beads. Also, the DSC outcomes demonstrate that Tc and Tm for electrospun PBS/CNT nanocomposite is greater than that of pure PBS which is about 6 °C and 3 °C, respectively. Moreover, the improvement in elasticity and strength can be credited to increase in a content of 3% MWCNT in the PBS matrix which in turn efficiently transfers the load homogeneously and uniformly. The inclusion of 3% MWCNT in the PBS matrix reduces tan δ and increases the storage modulus which also results in refinement of molecular chain relaxation. The fall in calculated tan δ enhances molecular relaxation, and aids PBS/MWCNT composites to absorb more energy. The addition of MWCNT in appropriate quantity improved electrical properties and EMI shielding characteristics for 3.0% PBS/MWCNTs nanocomposite. In addition, high SE_T_ of around 27.5 dB was also gained. The stability and durability of future EMI shielding materials can be achieved by combining adequate electromagnetic shielding and hydrophobicity. Finally, the results of our study will help to create biodegradable conductive polymer nanocomposite with exceptional EMI shielding qualities encouraging their use in the field of electromagnetic shielding materials.

## Figures and Tables

**Table 1:** Process parameters for PBS/MWCNT electrospun fiber.

Sample	Solvent Mixture Chloroform/Acetone (g)	PBS (g)	MWCNT (g)	Feed Rate mL/h	Needle-to-Collector Distance (cm)	Voltage (kV)
Neat PBS	12.5	2.500	-	0.5	11	20
PBS-0.5%MCNT	12.5	2.512	0.01256	0.5	11	20
PBS-1%MWCNT	12.5	2.513	0.02513	0.7	12	21
PBS-2%MWCNT	12.5	2.521	0.05042	0.8	12	22
PBS-3%MWCT	12.5	2.532	0.07596	0.8	12	22

**Table 2:** EDS results.

Sample	Weight% C	Weight% O	Atomic% C	Atomic% O
Neat PBS	60.02	39.98	66.67	33.33
0.5 wt% CNT/PBS	60.24	39.76	66.87	33.13
1.0 wt% CNT/PBS	61.45	38.55	68.06	31.94
2.0 wt% CNT/PBS	62.88	37.12	69.46	30.54
3.0 wt% CNT/PBS	63.31	36.69	70.85	29.15

**Table 3:** Thermal properties of PBS/MWCNT nanocomposites.

Specimen	T_C_ °C	ΔHc J/g	Tm °C	ΔH_m_ J/g	*X*_c_ %
Neat PBS	78.8	59.6	98.6	54.9	28.1
0.5%MCNT/PBS	80.1	61.3	99.1	56.3	28.6
1%MWCNT/PBS	81.5	62.9	101.6	57.9	28.9
2%MWCNT/PBS	82.7	63.3	102.8	57.2	29.4
3%MWCT/PBS	83.6	63.1	103.9	56.6	29.9

**Table 4:** Mechanical properties of PBS/MWCNT nanocomposite.

Specimen	Tensile Strength MPa	Elastic Modulus GPa	Elongation at Break %	Storage Modulus MPa	Tanδ
Neat PBS	1.81 ± 0.17	0.41 ± 0.02	115.15 ± 0.30	1312 ± 11	2.10 ± 0.03
0.5%MCNT/PBS	2.19 ± 0.18	0.48 ± 0.03	105.73 ± 0.22	1317 ± 13	1.79 ± 0.02
1%MWCNT/PBS	2.37 ± 0.15	0.56 ± 0.05	88.21 ± 0.31	1345 ± 13	1.63 ± 0.02
2%MWCNT/PBS	2.49 ± 0.17	0.65 ± 0.03	75.97 ± 0.23	1429 ± 15	1.57 ± 0.03
3%MWCT/PBS	2.61 ± 0.15	0.72 ± 0.02	61.51 ± 0.24	1458 ± 12	1.47 ± 0.02

## Data Availability

The original contributions presented in this study are included in the article. Further inquiries can be directed to the corresponding authors.
